# Association of *In Utero* Organochlorine Pesticide Exposure and Fetal Growth and Length of Gestation in an Agricultural Population

**DOI:** 10.1289/ehp.8423

**Published:** 2005-12-02

**Authors:** Laura Fenster, Brenda Eskenazi, Meredith Anderson, Asa Bradman, Kim Harley, Hedy Hernandez, Alan Hubbard, Dana B. Barr

**Affiliations:** 1 California Department of Health Services, Division of Environmental and Occupational Disease Control, Richmond, California, USA; 2 Center for Children’s Environmental Health Research, School of Public Health, University of California, Berkeley, California, USA; 3 Impact Assessment, Inc., Richmond, California, USA; 4 Natividad Medical Center Foundation, Salinas, California, USA; 5 Center for the Health Assessment of Mothers and Children of Salinas (CHAMACOS), Salinas, California, USA; 6 National Center for Environmental Health, Centers for Disease Control and Prevention, Atlanta, Georgia, USA

**Keywords:** birth weight, DDE, DDT, gestational age, hexachlorobenzene, organochlorine, pesticides, pregnancy, serum

## Abstract

From 1940 through the 1970s, organochlorine compounds were widely used as insecticides in the United States. Thereafter, their use was severely restricted after recognition of their persistence in the environment, their toxicity in animals, and their potential for endocrine disruption. Although substantial evidence exists for the fetal toxicity of organochlorines in animals, information on human reproductive effects is conflicting. We investigated whether infants’ length of gestation, birth weight, and crown–heel length were associated with maternal serum levels of 11 different organochlorine pesticides: *p*,*p*′-dichlorodiphenyltrichloroethane (*p*,*p*′-DDT), *p*,*p*′-dichlorodiphenyldichloroethylene (*p*,*p*′-DDE), *o*,*p*′-dichlorodiphenyltrichloroethane (*o*,*p*′-DDT), hexachlorobenzene (HCB), β-hexachlorocyclohexane (β-HCCH), γ-hexachlorocyclohexane (γ-HCCH), dieldrin, heptachlor epoxide, oxychlordane, *trans*-nonachlor, and mirex. Our subjects were a birth cohort of 385 low-income Latinas living in the Salinas Valley, an agricultural community in California. We observed no adverse associations between maternal serum organochlorine levels and birth weight or crown–heel length. We found decreased length of gestation with increasing levels of lipid-adjusted HCB (adjusted β= −0.47 weeks; *p* = 0.05). We did not find reductions in gestational duration associated with any of the other organochlorine pesticides. Our finding of decreased length of gestation related to HCB does not seem to have had clinical implications for this population, given its relatively low rate of preterm delivery (6.5%).

Organochlorine pesticides were widely used in the United States and worldwide from 1940 through the 1970s, but most have been eliminated or restricted in use after recognition of their persistence in the environment, bioaccumulation in animals and humans, and toxicity in laboratory animals and wildlife (United Nations Environment Programme 2002). For example, > 4 billion pounds of *p*,*p*′-dichlorodiphenyltrichloroethane (*p*,*p*′-DDT) were applied in agriculture and for mosquito and other insect control until it was banned for most uses in the United States in 1973. Hexachlorobenzene (HCB), a fungicide, was applied to grain seeds, such as wheat, from the 1940s to the late 1970s and was used in the United States until 1984 [Agency for Toxic Substances and Disease Registry (ATSDR) 2002]. HCB is still produced as a by-product or impurity in the manufacture of chlorinated organic chemicals, several pesticides, chlorine gas, and certain smelting operations and during the combustion of coal, with production estimates as high as 1 million pounds per year (ATSDR 2002). Technical hexachlorocyclohexane (HCCH), an insecticide, was widely used between 1940 and 1976 in the United States. The β-isomer of HCCH (β-HCCH) may be the most toxicologically significant because of its persistence and estrogenic effects (Li 1999). Although most organochlorine pesticides are no longer in use, many still persist in the environment and either continue to bioaccumulate or decrease slowly in humans.

Organochlorine pesticides may be endocrine disruptors (National Research Council 1999) and may also adversely affect fetal development. Maternal serum and umbilical cord blood levels of *p*,*p*′-DDT and/or *p*,*p*′-dichlorodiphenyldichloroethylene (*p*,*p*′-DDE), a metabolite of *p*,*p*′-DDT, have been associated in some studies with preterm birth (Longnecker et al. 2001; Ribas-Fito et al. 2002; Saxena et al. 1980; Wasserman et al. 1982), decreased birth weight (O’Leary et al. 1970; Weisskopf et al. 2005), or intrauterine growth retardation (Longnecker et al. 2001; Siddiqui et al. 2003). In other studies, increased levels of *p*,*p*′-DDT and/or *p*,*p*′-DDE in maternal serum, cord blood, or breast milk were not related to either decreased infant birth weight (Bjerregaard and Hansen 2000; Dewailly et al. 1993b; Gladen et al. 2003; Karmaus and Zhu 2004; Needham et al. 2005; Rogan et al. 1986) or preterm birth (Berkowitz et al. 1996; Needham et al. 2005; Torres-Arreola et al. 2003).

A small number of studies have examined the health consequences of other organochlorine pesticide exposure, with inconsistent findings in relationship to fetal growth outcomes. Higher HCB serum levels in preterm deliveries compared with term births were reported in one birth cohort study (Ribas-Fito et al. 2002) but not in a case–cohort study (Torres-Arreola et al. 2003). Additionally, Bjerregaard and Hansen (2000) did not find a significant association between HCB levels in cord blood and gestational duration. The literature on β-HCCH is also conflicting. One study reported a possible elevated risk for preterm delivery with increasing maternal serum β-HCCH levels (Torres-Arreola et al. 2003), whereas other studies did not find relationships between serum β-HCCH levels and measures of gestational duration or fetal growth (Gladen et al. 2003; Ribas-Fito et al. 2002; Siddiqui et al. 2003).

In the present study, we examined whether exposure to 11 different organochlorine pesticides, as assessed by measurements in maternal serum, was associated with shortened length of gestation and poorer fetal growth in a birth cohort from an agricultural community in California (Eskenazi et al. 2004). Most of the pregnant women were immigrants from Mexico, where they may have been directly exposed to organochlorine pesticides such as *p*,*p*′-DDT, which was restricted for malarial control use in 1991 and banned in 2000, as well as HCB and HCCH, which were used until the early 1990s (Chanon et al. 2003; Instituto de Salud Ambiente y Trabajo 1998; Li 1999).

## Materials and Methods

### Participants and recruitment.

The Center for the Health Assessment of Mothers and Children of Salinas (CHAMACOS) project, a component of the Center for Children’s Environmental Health Research at the University of California, Berkeley, is a longitudinal birth cohort study of the effects of pesticides and other environmental exposures on the health of pregnant women and their children living in the Salinas Valley, California. The study population and its materials and methods have been previously described in detail (Eskenazi et al. 2003, 2004). Between October 1999 and October 2000, pregnant women entering prenatal care at Natividad Medical Center, a county hospital located in the town of Salinas, or at five centers of Clinica de Salud del Valle de Salinas, were screened for eligibility and then recruited to participate in the longitudinal study. Women were eligible to participate if they were < 20 weeks gestation, ≥18 years of age, English or Spanish speaking, eligible for Medi-Cal, and planning to deliver at Natividad Medical Center. A total of 601 women were eligible and agreed to participate in the CHAMACOS study. After losses due to miscarriage, moving, or dropping from the study before delivery, birth weight information was available for 538 women and their neonates. Of these women, 421 had adequate serum stored for analysis of organochlorine levels. For this analysis, we excluded women with gestational or preexisting diabetes (*n* = 22), hypertension (*n* = 10), twin births (*n* = 2), or stillbirths (*n* = 2), resulting in a final sample size of 385. Written informed consent was obtained from all participants, and the study was approved by the institutional review boards of all collaborating organizations.

### Interview and medical record abstraction.

Bilingual, bicultural staff interviewed participants in English or Spanish during the first and second trimesters of pregnancy and again shortly after delivery. Questionnaire data collected included demographic information and lifestyle behaviors such as alcohol and tobacco consumption during the previous trimester. Information about previous pregnancies and any medical conditions, medications, or pregnancy complications was also obtained by interview and confirmed through medical records, which were abstracted by a registered nurse.

### Organochlorine exposure assessment.

Blood samples were collected by venipuncture from the mother at the time of the second pregnancy interview (*n* = 352; mean gestation ± SD = 26 ± 2.9 weeks) and in the hospital before delivery (*n* = 33). We measured the following 11 organochlorine pesticides, degradates, or metabolites in each serum sample—*p*,*p*′-DDT, *p*,*p*′-DDE, *o*,*p*′-DDT, β-HCCH, γ-hexachlorocyclohexane (γ-HCCH), dieldrin, HCB, heptachlor epoxide, oxychlordane, *trans*-nonachlor, and mirex—using gas chromatography/high-resolution mass spectrometry (GC/MS). In brief, we enriched 1 g of serum with isotopically labeled analogues of the target analytes. We lyophilized the serum to remove all traces of water then extracted the organochlorines using accelerated solvent extraction (10% dichloromethane in hexane). The extract was cleaned online with Florisil during the extraction process. We further purified the cleaned extract using gel permeation chromatography and then concentrated it for analysis. We analyzed the extracts using GC/MS (Barr et al. 2003). Quantification was achieved using isotope dilution calibration. We included quality-control materials and blank samples in each analytical run to ensure proper operation of the method. The average limits of detection (LODs) of the organochlorine pesticides were as follows: β-HCCH, 1.60 ± 0.70 pg/g; dieldrin, 19.90 ± 5.74 pg/g; γ-HCCH, 1.63 ± 0.79 pg/g; HCB, 0.85 ± 0.95 pg/g; heptachlor epoxide, 1.47 ± 0.65 pg/g; mirex, 0.73 ± 0.93 pg/g; *o*,*p*′-DDT, 1.32 ± 2.15 pg/g; oxychlordane, 2.28 ± 0.99 pg/g; *p*,*p*′-DDE, 2.98 ± 2.10 pg/g; *p*,*p*′-DDT, 1.58 ± 1.75 pg/g; and *trans*-nonachlor, 1.48 ± 0.91 pg/g. Data below the LOD were assigned the value of LOD/2, following Hornung and Reed (1990).

We measured total cholesterol and triglycerides using standard clinical enzymatic methods (Roche Chemicals, Indianapolis, IN). Total lipids were calculated using the summation method reported by Phillips et al. (1989). The laboratory and analytical methods were certified according to guidelines set forth in the Clinical Laboratory Improvement Amendment (U.S. Department of Health and Human Services 1988).

### Definition of outcomes.

We obtained infant birth weight and crown–heel length from hospital delivery logs and medical records. Gestational age was abstracted from the medical record and was based on ultrasound procedures for 25% of women. Because ultrasound estimates of gestational age may mask intrauterine growth retardation, we also estimated gestational age based on the woman’s self-reported date of last menstrual period. Results were similar using both methods; the results using the medical record gestational age are reported here. “Preterm delivery” was defined as birth at < 37 completed weeks of gestation. “Low birth weight” was defined as < 2,500 g. “Small for gestational age” was defined as below the 10th percentile for gestational age according to ethnicity (Mexican American or non-Hispanic white), parity, and infant sex from national data (Overpeck et al. 1999).

### Data analysis.

A total of 6.5% of the births (*n* = 25) were preterm, 2.9% (*n* = 11) of children had low birth weight, and 4.2% (*n* = 16) were small for gestational age. Because numbers for these dichotomous outcomes were small, we could not get reliable estimates from logistic regression. Therefore, we do not present any model results for dichotomous outcomes.

We first used localized weighted regression (LOESS) to produce smoothed plots of the continuous fetal growth outcomes and the 11 different organochlorine exposure levels in order to visually examine the data for nonlinear relationships (MathSoft Inc. 1997). We used linear regression models to test for associations between each of the serum organochlorine levels and length of gestation, birth weight, and crown–heel length. Models of birth weight and length were adjusted for gestational age and gestational age squared. We examined each organochlorine singly, rather than including other organochlorines in the models as potential confounders, in order to compare results with previous studies and because the levels of many organochlorines were highly correlated.

Organochlorine levels were lipid adjusted (nanograms per gram of lipid) and treated primarily as continuous variables on a log_10_ scale. We also ran all multivariate models with organochlorine levels reported in pico-grams per gram of serum, where serum tri-glycerides and cholesterol were entered as continuous variables in the model. Results were not appreciably different for these analyses, and we therefore chose to present results using the organochlorine levels on a lipid-adjusted basis. We also performed a series of regressions in which the organochlorine levels were dichotomized into categories of ≥75th percentile and < 75th percentile.

We examined potential confounders from the following risk factors reported in the literature: mother’s age, parity, marital status, family income, mother’s education, mother’s country of birth, prepregnancy body mass index (BMI), pregnancy weight gain, timing of entry into prenatal care, baby’s sex, caffeinated beverage intake, smoking, environmental tobacco smoke, maternal alcohol consumption, illicit drug use, bleeding during pregnancy, and mother’s work during pregnancy. The final models included risk factors associated with the outcomes at *p*-values ≤0.10 or risk factors known to be strongly associated with the outcomes from literature (e.g., maternal age, parity, family income, timing of entry into prenatal care, smoking). In models investigating length of gestation, we included metabolite levels of the organophosphate pesticide dimethyl phosphate, measured in the maternal urinary samples, because a strong association between decreased length of gestation and increasing dimethyl phosphate levels later in pregnancy was previously observed in the CHAMACOS cohort (Eskenazi et al. 2004). Similarly, we included levels of total dialkyl phosphates (DAPs) measured at 26 weeks in the models for birth weight and crown–heel length because of associations and literature reported by Eskenazi et al. (2004). Although levels of polychlorinated biphenyls (PCBs) in this population were low, we considered as a confounder a measure of total PCBs based on the formula developed by Needham et al. (2005) [(PCB-138 + PCB-153 + PCB-180) × 1.54]. The sum of the three major congeners is multiplied by 1.54 because Needham et al. (2005) have determined that the summed concentration of those congeners account for 65% of total PCBs (100%/65% = 1.54). The PCB levels were measured in the same serum samples as the organochlorine pesticides. Total PCBs were not retained in the final models because they were not independently related to the outcomes and did not alter the relationship between serum organochlorine levels and length of gestation or fetal growth. Finally, we conducted analyses that included an interaction term for sex and serum organochlorine levels. These analyses were prompted because organochlorines have been reported to affect males and females differently (Dewailly et al. 1993b). We did not observe any evidence of sex differences in our data, so these interaction terms were not included in final models.

The final models for length of gestation included continuous variables for maternal age, week prenatal care was initiated, and dimethyl phosphate in urine at 26 weeks, as well as categorical variables for parity, mother’s country of birth, family income, and smoking. Final models for birth weight and crown–heel length included all of the variables in models for length of gestation, except that total DAPs replaced dimethyl phosphate levels. In addition, the models for birth weight and crown–heel length included continuous variables for pregnancy weight gain, gestational age, and gestational age squared, as well as categorical variables for prepregnancy BMI and infant sex. Analyses were performed using SAS version 8.02 (SAS Institute Inc., Cary, NC) and S-Plus version 4.5 (Mathsoft Inc., Seattle, WA).

## Results

Table 1 summarizes the sociodemographic characteristics of the population. The women averaged 25.5 years of age (SD = 4.8), more than two-thirds were parous, 81% were married, and only 22% had graduated high school. Approximately 86% of the women were born in Mexico, with more than half residing in the United States for ≤5 years. About 63% of the women were living at or below poverty level, with almost all living below 200% of the poverty line. Prepregnancy BMI scores indicated that 60% of the women were overweight or obese before pregnancy. Very few women reported smoking (5.7%), illicit drug use (1.3%), or alcohol consumption (0.8%) during pregnancy, and more than two-thirds began prenatal care in the first trimester. During their pregnancy, approximately 28% of the women worked in the fields, another 12% had other agricultural jobs including packing shed, nursery, and greenhouse work, and 83% had agricultural workers living in their homes.

For each organochlorine, Table 2 shows the range of the LOD, the percentage of observations above the LOD, as well as the minimum, 10th percentile, median, 90th percentile, maximum, geometric mean, and 95% confidence interval (CI) for the geometric mean of the organochlorine level. Almost all serum samples had levels above the detection limit (ranging from 86 to 100% for the 11 organochlorine pesticides).

The mean (± SD) duration of gestation was 38.9 ± 1.5 weeks, the mean birth weight was 3477.0 ± 496.4 g, and the mean crown–heel length was 50.4 ± 2.5 cm. Table 3 shows the unadjusted regression results for organochlorine levels in relationship to length of gestation and fetal growth outcomes. Because models were run with organochlorines on the log_10_ scale, a unit of increase would mean a 10-fold increase in the organochlorine. None of the organochlorine levels was significantly related to a decrease in length of gestation. Increasing levels of HCB were associated with a nonsignificant decrease in length of gestation (β= −0.35 weeks; *p* = 0.12). We observed a somewhat lower birth weight associated with *p*,*p*′-DDE (β= −78 g; *p* = 0.09) (Table 3). We also noted a significant reduction in crown–heel length (β= −0.54 cm; *p* = 0.02) related to increasing *p*,*p*′-DDE levels (Table 3).

Table 4 presents the adjusted regression results for organochlorine levels and length of gestation, birth weight, and crown–heel length. After adjustment for covariates, the decrease in gestational length related to serum HCB levels became stronger. We found an approximately 0.47 week decrease in gestational age with each 10-fold increase in HCB (adjusted β= −0.47 weeks; 95% CI, −0.95 to −0.002; *p* = 0.05); this corresponds to about a 1 week decrease in gestational age across the range of exposure to HCB in our population. After adjustment, the relationships between *p*,*p*′-DDE levels and decreased birth weight and crown–heel length became weaker.

Examination of the LOESS plots did not indicate the presence of nonlinear associations. The results from analyses of the regressions where the organochlorine levels were dichotomized into categories of ≥75th percentile and < 75th percentile of exposure were consistent with the results from the multivariate linear regressions (data not shown). For example, the mean length of gestation was significantly shorter among women whose levels of HCB were in the upper quartile versus in the lower three quartiles (adjusted β= −0.55 weeks; 95% CI, −0.92 to −0.18; *p* = 0.004).

## Discussion

We examined the association between maternal serum levels of 11 different organochlorine pesticides and fetal growth and length of gestation in a population of primarily Mexican farmworker families living in an agricultural community in the United States (CHAMA-COS). The serum levels of certain organochlorines in our pregnant women, specifically *p*,*p*′-DDT, *p*,*p*′-DDE, and β-HCCH, were about 2-fold higher than those in Mexican-American women 18–40 years of age from the 1990–2000 National Health and Nutrition Examination Survey (NHANES) (Centers for Disease Control and Prevention 2005). Approximately 57% of the HCB levels in the CHAMACOS population were higher than the 57.4 ng/g LOD of the NHANES study, compared with only 3.4% for the NHANES population (Centers for Disease Control and Prevention 2005).

That HCB can produce reproductive toxicity in humans was established in studies of women in Turkey, where consumption of bread made from grain treated with HCB from 1955 through 1959 resulted in widespread poisoning, primarily manifested as porphyria cutanea tarda, in which damage to the liver results in disruption of heme synthesis and accumulation of porphyrins (Peters et al. 1982). The levels of HCB exposure in that population were presumably higher than any other population studied to date. Follow-up studies of porphyric mothers reported incidents of fetal deaths (Gocmen et al. 1989; Peters et al. 1982). A retrospective study conducted 40 years after the initial exposure found an increase in spontaneous abortions related to women living in the contaminated region or with porphyria cutanea tarda (Jarrell et al. 1998). None of these studies reported fetal growth measures.

We found an association between increasing maternal HCB serum levels and decreased length of gestation. Higher cord serum levels of HCB were found in preterm infants (*n* = 4) in a small birth cohort (*n* = 70) residing in an area in Spain contaminated with high levels of HCB (Ribas-Fito et al. 2002), whereas no association between HCB maternal serum levels and preterm births was found in a case–cohort study in Mexico City (Torres-Arreola et al. 2003). It is noteworthy that the serum HCB levels in mothers of premature infants in the Mexico City study were approximately half those of the CHAMACOS women (median, 44.9 vs. 100.7 ng/g lipid). Bjerregaard and Hansen (2000) examined the relationship of HCB measured in cord blood to gestational age and birth weight in a sample of approximately 120 births in the Disko Bay area in Greenland. After controlling for potential confounders in a linear model, they did not find a significant relationship between HCB levels and either gestational duration or birth weight; however, effect estimates were not presented, so our ability to make further comparisons is limited. A lack of association between HCB levels and birth weight was also found in the Spanish population (Ribas-Fito et al. 2002) and in a Ukrainian population (Gladen et al. 2003).

We did not find an HCB-associated decrease in crown–heel length such as that reported in the Spanish study (Ribas-Fito et al. 2002). To compare HCB levels in the two studies (cord vs. maternal serum), we assumed that maternal serum has approximately 2.5 times the lipid content of cord serum (Centers for Disease Control and Prevention, unpublished data; Waliszewski 2001). Using this conversion, we estimated that our HCB levels were lower than those in the Ribas-Fito study (median, 65 vs. 463 ng/g lipid), which may explain the discrepancy in results. Dewailly et al. (1993b) reported a significant decrease in crown–heel length of 40 male (*p* = 0.003) but not female Inuit infants associated with increasing HCB levels; these analyses were adjusted for age of the mother and length of the pregnancy. Using multivariate models on CHAMACOS data adjusted for the same covariates as Dewailly et al. (1993b), we found that a 10-fold increase in maternal serum levels of HCB was associated with a nonsignificant 0.30 cm decrease in crown–heel length for males (95% CI, −1.27 to 0.66). The mean HCB level for our entire sample (96 ng/g lipid in maternal serum; 95% CI, 85–106) was slightly lower than that reported by Dewailly et al. (1993a) (136 ng/g lipid in breast milk; 95% CI, 117–155).

Our results are similar to previous reports showing no association between increased levels of β-HCCH and decreased birth weight (Gladen et al. 2003) or intrauterine growth retardation (Siddiqui et al. 2003). Furthermore, increased levels of β-HCCH were not related to shortened length of gestation. Similarly, Ribas-Fito et al. (2002) did not observe higher levels of β-HCCH in cord serum of preterm births compared with term births. Although Torres-Arreola et al. (2003) detected a non-significant increased risk for preterm birth (odds ratio = 1.85; 95% CI, 0.95–3.66) associated with the highest tertile of maternal serum β-HCCH compared with the lowest tertile, the β-HCCH levels were somewhat higher than those in our study (median, 51.4 vs. 33.4 ng/g lipid, respectively).

When we examined the relationship between continuous *p*,*p*′-DDE levels and gestational duration in our study, we did not confirm the findings of Longnecker et al. (2001), who observed a significant relationship between increasing *p*,*p*′-DDE exposure and decreased gestational age in a U.S. birth cohort from the 1960s using a trend test. Lower *p*,*p*′-DDE levels, smaller samples size, and lower prevalence of preterm delivery in the CHAMACOS study are possible factors that contributed to our differing results. The median level of *p*,*p*′-DDE measured in the CHAMACOS population was lower than in the Longnecker et al. study population (1,004 vs. 4,274 ng/g lipid), as was the interquartile range (548–2,641 vs. 2,906–6,325 ng/g lipid). Additionally, the Longnecker et al. study population was larger (*n* = 2,380) and had a higher rate of preterm delivery (15%). Although we cannot rule out the possibility of real associations between *p*,*p*′-DDE exposure and adverse fetal growth outcomes, our CIs indicate the potential magnitude of these associations. For example, the 95% CI (−0.40 to 0.20) for the adjusted model of length of gestation and *p*,*p*′-DDE suggests that the true association is no greater than a 0.4-week reduction in gestational duration per 10-fold increase in *p*,*p*′-DDE.

To explore whether our findings are consistent with those reported by Longnecker et al. (2001), we adjusted for the same covariates and used the same categorization of *p*,*p*′-DDE levels (serum *p*,*p*′-DDE < 15 μg/L for the low exposure category and > 60 μg/L for the high exposure category). For each study, we calculated the adjusted mean difference in length of gestation between the high and low *p*,*p*′-DDE categories. We then calculated the difference in those mean differences between the two studies. CHAMACOS participants had on average a 0.39 week (95% CI, −1.26 to 0.48) smaller reduction in length of gestation than participants in the Longnecker et al. study. Given the width of the CI, we cannot assert that the results of the studies are consistent.

Our study had the following strengths: prospective design, data on several potential confounders, state-of-the-art laboratory techniques for measuring serum levels of organochlorines with low LODs, and a contemporary immigrant Mexican-American population with high levels of exposure. However, the low prevalence of adverse fetal growth outcomes in our population limited our ability to detect significant findings. The substantially lower rate of preterm delivery in the CHAMACOS cohort (6.5%), compared with that reported for Hispanic women of Mexican origin in the 1995 U.S. National Natality Survey (10.6%) (Matthews et al. 1998), suggests that HCB exposure had little clinical impact on length of gestation.

In summary, we did not find any adverse association of *in utero* organochlorine pesticide exposure with birth weight or crown–heel length using maternal serum measures to estimate exposure. We did find that exposure to the fungicide HCB was significantly related to a decrease in length of gestation, although it is possible that this finding is spurious because of multiple comparisons. HCB is no longer manufactured but is still produced as a by-product or impurity in the manufacture of chlorinated solvents and several pesticides currently in use (ATSDR 2002). Thus, because HCB may continue to enter the environment, its potential reproductive toxicity may remain a concern for human populations.

## Figures and Tables

**Table 1:** Demographic characteristics of CHAMA-COS participants, Salinas Valley, California (*n* = 385).

Characteristic	No. (%)
Mother’s age (years)	
18–24	181 (47.0)
25–29	127 (33.0)
30–34	53 (13.8)
≥35	24 (6.2)
Parity	
0	121 (31.4)
≥1	264 (68.6)
Mother’s education	
< 7th grade	161 (41.8)
7th–12th grade	139 (36.1)
Completed high school	85 (22.1)
Mother’s country of birth	
Mexico	330 (85.7)
United States	46 (12.0)
Other	9 (2.3)
Family income	
≤Poverty level	224 (62.6)
> Poverty level	134 (37.4)
Pregnancy weight gain (pounds)	
< 25	124 (32.3)
25–35	141 (36.7)
> 35	119 (31.0)
Mother’s BMI (kg/m^2^)	
Underweight and normal weight (< 18.5–24.9)	148 (39.7)
Overweight (25–29.9)	149 (39.9)
Obese (≥30)	76 (20.4)
Smoked during pregnancy	
Yes	22 (5.7)
No	363 (94.3)
Drank alcohol during pregnancy	
Yes	3 (0.8)
No	369 (99.2)
Trimester when prenatal care began	
First	259 (67.3)
Second	125 (32.5)
Third	1 (0.2)
History of low birth weight or preterm births	
Nulliparous	121 (35.4)
Neither	179 (52.3)
History of preterm births but not low birth weight	13 (3.8)
History of low birth weight but not preterm births	15 (4.4)
History of both	14 (4.1)
Mother’s work status during pregnancy	
Not working	146 (38.6)
Working in fields	106 (28.1)
Other agricultural work	47 (12.4)
Other work (not agricultural)	79 (20.9)

**Table 2:** Range of LOD, percent detectable, percentiles, and geometric means and 95% CIs of organochlorines measured in serum during pregnancy (CHAMACOS study, Salinas Valley, CA, 2000–2001).

Marker of exposure (ng/g lipid)	No.	LOD range	Percent > LOD	Minimum	10th percentile	Median	90th percentile	Maximum	Geometric mean	95% CI
*p*,*p*′-DDE	385	0.06 to 4.83	100.0	48.8	364.0	1003.7	8417.2	159303.3	1363.0	1198.1 to 1551.0
*p*,*p*′-DDT	385	0.06 to 4.70	100.0	1.6	4.5	12.1	273.8	33174.0	20.6	17.3 to 24.5
*o*,*p*′-DDT	383	0.04 to 6.05	95.6	0.07	0.39	1.2	10.5	1878.1	1.6	1.4 to 1.9
HCB	385	0.02 to 1.00	100.0	8.1	23.0	64.8	192.8	841.0	66.4	61.1 to 72.1
β -HCCH	383	0.05 to 1.28	99.7	0.07	5.3	37.2	127.4	2491.6	32.1	28.4 to 36.3
γ-HCCH	378	< 0.01 to 1.55	93.9	0.003	0.49	1.0	2.5	42.0	0.9	0.84 to 1.0
Dieldrin	358	0.90 to 6.62	90.8	0.0	2.6	5.8	11.5	104.0	5.7	5.3 to 6.1
Heptachlor epoxide	367	0.05 to 0.45	99.5	0.05	2.2	4.8	12.7	60.1	5.1	4.7 to 5.5
Oxychlordane	365	0.08 to 0.80	97.5	0.0	1.7	6.0	18.7	75.5	5.8	5.2 to 6.4
*trans*-Nonachlor	385	0.05 to 1.49	100.0	1.8	3.8	8.0	19.4	89.1	8.2	7.7 to 8.8
Mirex	384	0.01 to 0.69	85.9	0.04	0.13	0.29	1.2	15.9	0.3	0.31 to 0.38

**Table 3:** Crude association of organochlorines in maternal blood during pregnancy with length of gestation and fetal growth (CHAMACOS study, Salinas Valley, CA, 2000–2001).

	Length of gestation (weeks; *n* = 385)			Birth weight (g; *n* = 385)			Crown–heel length (cm; *n* = 381)		
Organochlorine (ng/g lipid, log_10_ scale)	β	95% CI	*p*-Value	β	95% CI	*p*-Value	β	95% CI	*p*-Value
*p*,*p*′-DDE	−0.05	−0.33 to 0.23	0.73	−78	−167 to 11	0.09*	−0.54	−1.00 to −0.09	0.02**
*p*,*p*′-DDT	0.11	−0.10 to 0.32	0.30	−33	−99 to 33	0.32	−0.26	−0.60 to 0.08	0.13
*o*,*p*′-DDT	0.16	−0.08 to 0.39	0.19	−23	−98 to 52	0.54	−0.15	−0.54 to 0.23	0.43
HCB	−0.35	−0.78 to 0.08	0.12	−96	−235 to 42	0.17	−0.25	−0.96 to 0.46	0.49
β -HCCH	0.14	−0.15 to 0.43	0.35	63	−31 to 157	0.19	0.14	−0.35 to 0.62	0.57
γ-HCCH	0.12	−0.23 to 0.46	0.51	43	−67 to 153	0.44	0.19	−0.37 to 0.74	0.51
Dieldrin	−0.24	−0.83 to 0.36	0.43	−0.8	−194 to 192	0.99	−0.01	−0.99 to 0.97	0.99
Heptachlor epoxide	−0.13	−0.61 to 0.36	0.61	82	−71 to 235	0.29	0.40	−0.37 to 1.18	0.31
Oxychlordane	−0.06	−0.41 to 0.30	0.75	58	−57 to 173	0.32	0.25	−0.33 to 0.83	0.39
*trans*-Nonachlor	−0.09	−0.65 to 0.47	0.76	17	−164 to 198	0.86	0.33	−0.59 to 1.25	0.48
Mirex	−0.12	−0.49 to 0.26	0.54	−87	−207 to 33	0.15	−0.39	−1.01 to 0.22	0.21

* *p* < 0.10;

** *p* < 0.05.

**Table 4:** Adjusted association of organochlorines in maternal blood during pregnancy with length of gestation and fetal growth (CHAMACOS study, Salinas Valley, CA, 2000–2001).

	Length of gestation^a^ (weeks; *n* = 340)			Birth weight^b^ (g; *n* = 330)			Crown–heel length^b^ (cm; *n* = 326)		
Organochlorine (ng/g lipid, log_10_ scale)	β	95% CI	*p*-Value	β	95% CI	*p*-Value	β	95% CI	*p*-Value
*p*,*p*′-DDE	−0.10	−0.40 to 0.20	0.51	−46	−129 to 37	0.28	−0.37	−0.83 to 0.09	0.11
*p*,*p*′-DDT	0.04	−0.18 to 0.26	0.72	−32	−93 to 29	0.30	−0.25	−0.58 to 0.09	0.15
*o*,*p*′-DDT	0.07	−0.18 to 0.32	0.58	−18	−86 to 50	0.61	−0.13	−0.51 to 0.25	0.50
HCB	−0.47	−0.95 to −0.002	0.05*	−23	−154 to 108	0.73	0.07	−0.66 to 0.80	0.85
β -HCCH	0.07	−0.30 to 0.44	0.71	25	−74 to 125	0.62	0.01	−0.54 to 0.57	0.97
γ-HCCH	0.02	−0.33 to 0.36	0.92	20	−71 to 112	0.66	0.06	−0.45 to 0.56	0.82
Dieldrin	−0.49	−1.14 to 0.16	0.14	18	−164 to 201	0.84	0.08	−0.92 to 1.08	0.87
Heptachlor epoxide	−0.15	−0.71 to 0.42	0.61	44	−105 to 194	0.56	0.25	−0.58 to 1.07	0.56
Oxychlordane	−0.03	−0.40 to 0.35	0.89	64	−39 to 168	0.22	0.37	−0.19 to 0.93	0.20
*trans*-Nonachlor	−0.08	−0.70 to 0.54	0.79	38	−128 to 204	0.65	0.53	−0.38 to 1.45	0.25
Mirex	0.11	−0.31 to 0.54	0.60	−18	−131 to 94	0.75	−0.11	−0.73 to 0.51	0.72

a Models adjusted for maternal age, parity, country of birth, family income, timing of entry into prenatal care, smoking, and total dimethyls in urine at 26 weeks.

b Models adjusted for maternal age, parity, country of birth, pregnancy weight gain, prepregnancy BMI, family income, timing of entry into prenatal care, smoking, total DAPs in urine at 26 weeks, infant sex, gestational age, and gestational age squared.

* *p* < 0.10.
